# Clinical Characteristics Associated With Very Preterm Delivery Despite Transabdominal Cerclage: A Cohort Study

**DOI:** 10.1111/1471-0528.70177

**Published:** 2026-02-16

**Authors:** Laura van der Krogt, Lisa Story, Natalie Suff, Andrew Shennan

**Affiliations:** ^1^ Division of Women’s Health King’s College London, St Thomas’ Hospital London UK

**Keywords:** cerclage failure, cervical length, clinical research, preterm birth, preterm labour, transabdominal cerclage

## Abstract

**Objective:**

To identify factors associated with unsuccessful transabdominal cerclage (TAC) insertion, defined as delivery before 32 weeks, including maternal demographics, preterm birth risk factors, operative details (laparoscopic or open, pre‐pregnancy or during pregnancy) and subsequent cervical length.

**Design:**

Observational cohort study.

**Setting:**

A tertiary‐level London hospital.

**Sample:**

Women who underwent TAC between 2011 and 2024 and consented to inclusion in the Preterm Clinical Network (PCN) Database.

**Methods:**

Data were collected from the PCN database and maternity records.

**Main Outcome Measures:**

Characteristics of women delivering before versus after 32 weeks were compared using student's *t*‐test where data were continuous and chi‐squared where data were categorical (SPSS version 29.0).

**Results:**

Among 125 pregnancies following TAC, eight (6.4%) delivered before 32 weeks. Previous trachelectomy was strongly associated with preterm delivery (*p* < 0.0001). Mean cervical length differed significantly between groups: first trimester (19.3 mm vs. 33.8 mm, *p* < 0.001) and second trimester (18.8 mm vs. 31.9 mm, *p* < 0.001). Of 30 women with prior pregnancies using the same TAC, 80% (24/30) achieved at least one term birth and 13.3% (4/30) had two or more term deliveries.

**Conclusion:**

TAC is highly effective, with over 90% of women delivering beyond 32 weeks. Factors linked to unsuccessful TAC include prior trachelectomy and short cervical length in early pregnancy. Effectiveness persists across subsequent pregnancies, with many women achieving multiple term births using the same TAC.

## 1 Introduction

Preterm birth (PTB), defined as delivery before 37 weeks completed gestation, remains an important global issue affecting approximately 10% of births worldwide [1]. Prematurity is associated with significant mortality and morbidity, representing the leading cause of infant death under the age of five [1]. Outcomes are inversely correlated with gestational age, with those infants born at extremely and very preterm gestations (between 24 and 32 weeks) being at greatest risk [1, 2, 3, 4].

Cervical cerclage, the placement of a stitch into the cervical tissue, is one of the main interventions for the prevention of preterm birth [5]. The exact mechanism of action of cerclage is unknown, but it may act by providing mechanical support to the cervix and/or act as a barrier to ascending infection, by maintaining cervical length and the mucous plug [6]. Several different types of cerclage procedures exist, including transvaginal and transabdominal cerclage.

The majority of cerclages performed are inserted vaginally during pregnancy. However, evidence indicates that in women for whom a previous transvaginal cerclage was unsuccessful, a transabdominal cerclage (TAC) may be superior and most TACs are performed for this indication [7, 8]. Other groups who may be offered TAC are those who have had extensive cervical surgery such as trachelectomy or multiple large loop excisions of the transformation zone [9, 10]. Women with a history of preterm birth or mid‐trimester loss following a full dilatation caesarean section (FDCS) may also benefit from a TAC [9, 11].

A TAC is placed abdominally at the level of the cervico‐isthmic junction and is consequently higher in the cervix than vaginally placed cerclages. It can be inserted laparoscopically or at laparotomy and can be placed pre‐pregnancy or in the first trimester [7]. TAC is associated with high rates of live births, with reported success rates ranging from 80% to 90% [8, 12, 13, 14] compared with 75%–80% for transvaginal cerclage [8, 15]. A recent meta‐analysis further suggests that transabdominal cerclage may outperform transvaginal cerclage in reducing perinatal mortality and preterm birth rates in patients at high risk of preterm birth [16]. However, the ability to predict which TACs are likely to be unsuccessful remains limited. This information would allow risk stratification of women with TAC and may highlight those who would benefit from closer surveillance following TAC.

This study therefore aims to investigate the factors associated with an unsuccessful transabdominal cerclage insertion. In line with existing literature, including the MAVRIC study, an unsuccessful cerclage was defined as delivery before 32 weeks [8]—a clinically meaningful threshold given the substantially higher morbidity and mortality associated with very preterm birth [2, 3]. Parameters assessed included maternal demographics, risk factors for preterm birth, operative details and cervical length.

## 2 Materials and Methods

A retrospective cohort study was performed of women who received a transabdominal cerclage at a tertiary‐level London hospital and had consented to inclusion in the Preterm Clinical Network (PCN) Database from 2011 to 2024 (research ethics committee reference number: 16/ES/0093).

Maternal demographic data including age, BMI, ethnicity and parity were collected. Risk factors for preterm birth were recorded, such as previous preterm birth, previous mid‐trimester loss, previous cervical surgery and previous unsuccessful transvaginal cerclage. Operation details were recorded when available from corresponding clinical records—this included whether the procedure was open or laparoscopic and performed in pregnancy or pre‐pregnancy. Transvaginal ultrasound measurements of cervical length and gestational age at the time of scanning were collected across the first, second and third trimesters. Pregnancy outcomes including gestation at delivery, admission to the neonatal unit and maternal length of stay in hospital were recorded. If women had more than one pregnancy with a transabdominal cerclage, details from the previous pregnancies were also collected.

Differences in characteristics between women who had a ‘successful’ TAC, (delivery > 32 weeks' gestation) and those who had an ‘unsuccessful’ TAC (delivery < 32 weeks' gestation) were analysed. Statistical analysis was conducted using student's *t*‐test for normally distributed continuous variables and chi‐squared for categorical variables (SPSS version 29.0).

## 3 Results

There were 125 pregnancies suitable for analysis. Maternal demographics including age, body mass index, ethnicity and parity were similar across the two groups. See Table 1 for a summary of maternal demographics.

**TABLE 1:** Maternal demographics and risk factors for preterm birth.

	Successful TAC *n* = 117 (%)	Unsuccessful TAC *n* = 8 (%)	*p*
Mean age, years (SD)	37 (5)	36 (5)	0.058
Mean body mass index, kg/m^2^ (SD)	28 (6)	24 (7)	0.119
Ethnicity			
Asian	14 (11.9)	2 (25.0)	0.325
Black	46 (39.3)	0 (0)	
Other	1 (0.9)	0 (0)	
Unrecorded	3 (2.6)	0 (0)	
White	53 (45.3)	6 (75.0)	
Parity			
0	36 (30.7)	5 (62.5)	0.461
1	50 (42.7)	2 (25.0)	
2	23 (19.7)	1 (12.5)	
3	6 (5.1)	0 (0)	
≥ 4	2 (1.7)	0 (0)	
Previous sPTB	45 (38.5)	3 (37.5)	0.957
Previous mid‐trimester loss^a^	77 (65.8)	2 (25.0)	0.152
Previous PPROM	31 (26.5)	4 (50.0)	0.152
Previous cervical surgery	44 (37.6)	5 (62.5)	0.163
Previous LLETZ/cone biopsy	36 (30.8)	0 (0)	0.166
Previous trachelectomy	5 (4.3)	5 (62.5)	< 0.001
Previous in‐labour CS	17 (14.5)	0 (0)	0.246
Previous FDCS	10 (8.5)	0 (0)	0.389
Uterine anomaly	3 (2.6)	0 (0)	0.647
Previous failed TVC	66 (56.4)	2 (25)	0.084
Previous pregnancy with TAC	30 (25.6)	1 (12.5)	0.405

*Note:* Table 1: Maternal demographics and risk factors for preterm birth in the study population.

Abbreviations: FDCS, full dilatation caesarean section; PPROM, preterm pre‐labour rupture of membranes; SD, standard deviation; sPTB, spontaneous preterm birth delivery < 37 weeks gestation.

^a^ Mid‐trimester loss—delivery between 14 to 24 weeks gestation.

Risk factors for preterm birth were similar across both groups; however, those with a history of previous trachelectomy were more likely to deliver less than 32 weeks (*p* < 0.0001). Of note, some women had more than one risk factor for preterm birth. See Table 1 for a summary of risk factors for preterm birth.

Operative details were available for 54 cases (43.2%). The majority of those for who the records were available, had an open TAC performed via laparotomy (51/54; 94.4%). Of the open TACs 5/51 (9.8%) delivered before 32 weeks. All the laparoscopic TACs delivered after 32 weeks (3/3; 100.0%). One of the TACs was done in the index pregnancy (1/125, 0.8%) at 12 weeks' gestation and the rest were done pre‐conception (124/125, 99.2%). However, when reviewing women who had a previous pregnancy with a TAC, it was identified that three additional TACs had initially been performed in‐pregnancy at gestational ages from 8 to 13 weeks. All four in‐pregnancy TACs resulted in a term delivery. See Table 2 for a summary of operative details.

**TABLE 2:** Operative details and transvaginal scan findings.

	Successful TAC	Unsuccessful TAC	*p*
	*n* = 117 (%)	*n* = 8 (%)	
TAC Type^a^			
Open TAC	46 (93.9)	5 (100)	0.416
Laparoscopic TAC	3 (6.1)	0 (0)	
TAC Timing^b^			
In pregnancy	1 (0.8)	0 (0)	
Pre‐pregnancy	116 (92.8)	8 (100)	
Mean first trimester CL in mm (SD)	33.8 (5) [*n* = 20]	19.3 (7) [*n* = 4]	< 0.001
Mean second trimester CL in mm (SD)	31.9 (9) [*n* = 108]	18.8 (14) [*n* = 7]	< 0.001
Mean third trimester CL in mm (SD)	34.1 (10) [*n* = 79]	26 (3) [*n* = 2]	0.260

*Note:* Table 2: Operative details and transvaginal ultrasound scan findings in different gestations in the study population.

Abbreviations: CL, cervical length; SD, standard deviation.

^a^ Perative details available for 54 cases (43.2%); *n* = 49 for successful TAC and *n* = 5 for unsuccessful TAC.

^b^ In the index case (note 3 patients had a prior pregnancy with TAC, where it was sited during that pregnancy).

Transvaginal ultrasound measurements of cervical length were recorded when performed at the preterm surveillance clinic. It was variable when these scans were performed, with 24 (19.2%) having a first trimester cervical length, 115 (92.0%) a second trimester cervical length and 81 (64.8%) a third trimester cervical length. There was a difference in the mean cervical length in the first and second trimesters between those who delivered less than 32 weeks and those who delivered after 32 weeks (19.3 mm versus 33.8 mm in the first trimester, *p* < 0.001 and 18.8 mm versus 31.9 mm in the second trimester, *p* < 0.001). There was no significant difference between third trimester cervical length between the groups (26 mm vs 34.1 mm, *p* = 0.260). See Table 2 for a summary of transvaginal scan findings.

Eight pregnancies (6.4%) delivered before 32 weeks. Of the deliveries less than 32 weeks, 5 (62.5%) had preterm prelabour rupture of membranes and evidence of chorioamnionitis and 1 (12.5%) had placental abruption and uterine rupture; there were no details available for the other 2 (25.0%). All deliveries were by caesarean section, except for one vaginal delivery at 16 weeks' gestation.

All those who delivered more than 32 weeks had a live birth. There was one mid‐trimester loss and two neonatal deaths in the group who delivered less than 32 weeks. Those who delivered less than 32 weeks were more likely to have a baby who was admitted to NICU (6/8, 75.0% vs. 14/117, 11.9%; *p* < 0.001). There was a difference in mean length of maternal postnatal stay between the two groups (3 days vs. 5 days, *p* = 0.01). See Table 3 for a summary of neonatal and postnatal outcomes.

**TABLE 3:** Neonatal and postnatal outcomes.

	Successful TAC *n* = 117 (%)	Unsuccessful TAC *n* = 8 (%)	*p*
Pregnancy outcome			< 0.001
Mid‐trimester loss	0 (0)	1 (12.5)	
Livebirth	117 (100)	5 (62.5)	
Neonatal death	0 (0)	2 (25.0)	
NICU admission			< 0.001
Yes	14 (11.9)	6 (75.0)	
No	100 (85.5)	2^a^ (37.5)	
Not documented	3 (2.6)	0 (12.5)	
Duration maternal postnatal stay (days)	3 (*n* = 106)	5 (*n* = 7)	0.011

*Note:* Table 3: Postnatal outcomes for women and neonates.

Abbreviation: NICU, neonatal intensive care unit.

^a^ These infants were not admitted to NICU as they passed away prior to admission.

**TABLE 4:** Case summaries for the unsuccessful cerclages.

	Risk factors for preterm birth	History of TVC	Timing of TAC	Open or laparoscopic	Previous pregnancy with TAC	Cervical length (by trimester)	GA at delivery	Delivery details	Outcome
1	Previous trachelectomy	No	Pre‐pregnancy	Open	No	1st–20 mm	31 + 4	EMCS‐PPROM and chorioamnionitis	Livebirth
2	Previous trachelectomy	No	Pre‐pregnancy	Open	No	2nd–31 mm, 3rd–28 mm	31 + 1	EMCS in labour	Livebirth
3	Previous trachelectomy	No	Pre‐pregnancy	Not available	No	2nd–34 mm, 3rd—33 mm	30 + 2	EMCS‐PPROM and chorioamnionitis	Livebirth
4	Previous MTL, previous PPROM	No	Pre‐pregnancy	Open	No	2nd–3 mm	26 + 6	EMCS‐chorioamnionitis	Livebirth
5	Previous MTL, previous sPTB	Yes	Pre‐pregnancy	Open	No	2nd–29 mm	26 + 4	EMCS–placental abruption and uterine rupture	NND
6	Previous trachelectomy, previous PPROM	No	Pre‐pregnancy	Not available	Yes–early miscarriage and 30/40 delivery	1st–12 mm, 2nd—4 mm	24 + 0	EMCS—PPROM and chorioamnointis	Livebirth
7	Previous trachelectomy	No	Pre‐pregnancy	Open	No	2nd–21 mm	24 + 4	EMCS–PPROM and chorioamnionitis	NND
8	Previous MTL	Yes	Pre‐pregnancy	Open	No	1nd–29 mm, 2nd–43 mm	16 + 1	Spontaneous vaginal delivery	MTL

*Note:* Table 4: Transvaginal ultrasound scan cervical length findings in different gestations in the study population.

Abbreviations: CL, cervical length; EMCS, emergency caesarean section; MTL, mid‐trimester loss; NND, neonatal death; PPROM, preterm prelabour rupture of membranes; sPTB, spontaneous preterm birth.

There were 30 women who had at least one pregnancy prior to the index case. Of these women 80.0% (24/30) had at least one prior term delivery and 13.3% (4/30) had two or more prior term deliveries with the transabdominal cerclage in situ. Four women had more than one pregnancy outcome (i.e., three term deliveries followed by one early miscarriage).

The subsequent pregnancies of the eight which ended less than 32 weeks were reviewed. One patient delivered less than 32 weeks in two pregnancies—first delivery at 30 weeks and second delivery at 24 + 4 weeks, with no further pregnancies documented. Two patients had no further pregnancies documented. One patient, who had a placental abruption and uterine rupture, had a hysterectomy at the time of delivery and was advised to consider adoption or surrogacy. One patient had an ongoing pregnancy at 30 weeks' gestation at the time of review. The final two patients had no hospital numbers and therefore records were not reviewed.

## 4 Discussion

### 4.1 Main Findings

In the small proportion of TACs that are unsuccessful (delivery < 32 weeks), there is an association with previous trachelectomy (*p* < 0.001) and short cervical length in the first and second trimesters (*p* < 0.001). Most unsuccessful cases were linked to PPROM and chorioamnionitis (5/8, 62.5%). This large cohort of over 120 pregnancies is among the first to explore factors associated with TAC failure. Overall, TAC remains highly effective, with > 90% achieving live birth beyond 32 weeks. Reassuringly, among women with a prior pregnancy using the same TAC, 80% (24/30) had at least one term birth, and 13.3% (4/30) had two or more term deliveries (Figure S1 and Table S1).

### 4.2 Interpretation

This study adds to the existing literature describing the efficacy of TAC, with reports of TAC success rates between 80% to 90% [8, 12, 14]. Most of the evidence consists of retrospective observational trials and meta‐analyses. It must be noted that there is no consistent definition of ‘success’ between these studies, with some using gestational ages of greater than 24, 32 weeks, 34 and 36weeks [17, 18, 19, 20, 21, 22, 23, 24]. The findings in this study align with the MAVRIC study, a multi‐centre randomised control trial comparing TVC and TAC in women with previous unsuccessful TVC. MAVRIC reported that 92.0% of women allocated a TAC delivered at more than 32 weeks [8]. In addition, 32 weeks was chosen in this study as a clinically relevant endpoint—as infants born at extremely and very preterm gestations are at greatest risk of morbidity and mortality.

Cerclage placement during trachelectomy is routinely practiced and currently recommended, with the cerclage placed at the level of the isthmus, creating a neocervix [25, 26]. However, there have been concerns raised about the efficacy of these cerclages as the anatomic supports that keep the TAC from migrating have likely been transected or removed as part of the procedure [13]. Indeed, a case series published by Burger et al. reports on 10 patients with displaced cerclages and associated obstetric and fertility complications including PPROM and mid‐trimester loss [27]. In this study, history of trachelectomy was associated with an unsuccessful TAC and delivery less than 32 weeks. In the women with a previous trachelectomy and unsuccessful TAC, 4 of 5 (80.0%) were due to PPROM and chorioamnionitis. In this cohort, the cervix is inherently short, and despite the TAC, there may not be a sufficient barrier to prevent ascending infection, a major cause of preterm labour. There was no documented cerclage displacement or need for repeat cerclage in these women.

The association between short cervical length in the first and second trimester and delivery before 32 weeks may be accounted for by the large proportion of women with trachelectomy in the unsuccessful TAC group, who would have a shorter cervical length to begin with. However, due to the small numbers of unsuccessful TAC in this group, it was not possible to adequately adjust for this or other potential confounders. The timing of cervical length within the group was also variable with only 24 (19.2%) having a first trimester cervical length, 115 (92.0%) a second trimester cervical length and 81 (64.8%) a third trimester cervical length.

This variability is likely caused by a lack of guidance for sonographic surveillance following cerclage, especially TAC [7]. Local guidance at the study site suggests CL should be performed once in the second and third trimesters. Therefore, a very small number of included women had a CL performed in the first trimester and these findings should be interpreted with caution. The fact that there was no difference in third trimester CL is likely to be explained by the fact that most of the unsuccessful TACs, 5/8 (62.5%), delivered before the third trimester and did not have a cervical length performed during this gestational period. Again, the findings are likely attributable to the small number included and rarity of events.

However, a study examining longitudinal cervical lengths demonstrated that with TACs the cervix was more likely to remain long and stable [28]. It was demonstrated that cervical length measurement after 20 weeks was a good predictor of PTB less than 32 weeks in women with TAC [28]. This suggests there is a value in performing a cervical length scan in women with a TAC.

Women can have more than one term pregnancies with a cerclage in situ [14, 29, 30]. In keeping with previous studies, in this cohort 30 women had a pregnancy prior to the index case. Of these, 80.0% (24/30) had at least one term delivery and 13.3% (4/30) had two or more term deliveries. There was no association with previous pregnancy with the TAC and TAC ‘failure’ in the index pregnancy.

It should be recognised that having a TAC in situ requires delivery by caesarean section. This means having multiple abdominal surgeries—to place the TAC and for delivery of all future pregnancies. Moreover, managing mid‐trimester loss in the context of TAC is complex. Reports suggest that up to 18 weeks' gestation surgical management may be performed through the TAC or the TAC can be removed via anterior or posterior colpotomy [31, 32, 33]. If this is unsuccessful, a hysterotomy may be required [32].

This work adds to the existing literature and can be used clinically to counsel patients regarding TAC. Reassurance can be given to patients that having more than one pregnancy with a TAC does not impact the likelihood of delivering greater than 32 weeks. Indeed, in this cohort many women had at least one and up to three term deliveries preceding the index pregnancy with the same TAC. It also suggests that women with a history of trachelectomy and those with short cervical length in the first and second trimester may benefit from additional monitoring as they are more likely to deliver less than 32 weeks. Moreover, the findings indicate that cervical length can be used to determine those who may deliver earlier despite TAC. Given the challenges in managing preterm birth in this group and the need for operative delivery, this is of added value.

Robust prospective and randomised studies are needed to clearly answer the questions regarding optimal surgical techniques and which patients are likely to benefit most from a TAC procedure. The NORACT study [NCT06122506], a prospective randomised control trial, is due to commence and aims to compare vaginal and laparoscopic abdominal cerclage. The ABOVE study pregnancy [ISCRTN 1097796], a prospective multicentre randomised control trial, compares transabdominal and transvaginal cerclage specifically in women with previous in‐labour caesarean section and mid‐trimester loss or preterm birth in a subsequent pregnancy. This trial also aims to compare pre‐conception and in‐pregnancy transabdominal cerclage in this group.

It must be recognised that designing such trials presents inherent complexities. Therefore, a prospective surgical register for TAC procedures would offer a robust platform for generating evidence. In the UK, the Obstetric Surveillance Survey aims to collect information on TACs performed including subsequent management and outcomes and is due to report [34].

### 4.3 Strengths and Limitations

This study report on one of the largest cohorts of TAC and among the few to identify factors specifically associated with TAC ‘failure’ [17, 23]. The retrospective design introduces inherent limitations including reliance on incomplete clinical data records, limited availability of operative details and reduced ability to adjust for unmeasured confounding.

The limited operative information is due to several factors. Some women who consent to the PCN database do not consent for review of their medical records, and some participants had their procedures at other institutions. Consequently, differences between laparoscopic and open techniques could not be assessed, though previous studies indicate comparable outcomes [21, 22, 35]. Similarly, there were few in pregnancy TAC cases, which limited the ability to analyse the timing of TAC. However, existing evidence indicates similar efficacy for pre‐conception and in‐pregnancy TAC [20, 21].

The small number of unsuccessful TAC cases reduced statistical power and limited the ability to adjust adequately for confounding. This particularly affects interpretation of associations involving cervical length, as a disproportionately high proportion of women in the unsuccessful TAC group had a prior trachelectomy and therefore began pregnancy with structurally shorter cervices. While this likely contributed to the observed associations, the sample size prevented more robust stratified or multivariable analyses.

Overall, interpretation of *p*‐values in observational studies is limited by the potential for residual confounding and the inability to infer causality. Again, given the small number of unsuccessful TAC cases, *p*‐values are presented as descriptive indicators of group differences rather than definitive evidence of association. These findings are therefore exploratory and hypothesis‐generating.

Finally, this analysis focused on deliveries before and after 32 weeks. While moderate‐to‐late preterm births (between 32 and 37 weeks) carry fewer risks than very preterm births (less than 32 weeks), they still pose significant risks for short and long‐term health [1, 36]. Future research should explore TAC outcomes across all gestational ages under 37 weeks to better inform clinical decision‐making.

## 5 Conclusion

TAC is a highly effective procedure, with over 90% of women achieving a livebirth beyond 32 weeks gestational age. A history of previous trachelectomy and short cervical length in the first and second trimester are associated with unsuccessful TAC leading to delivery before 32 weeks. Subsequent pregnancies with the same TAC remain effective, with many women achieving multiple term deliveries. These findings support counselling, risk stratification and tailored monitoring for women with TAC.

## Author Contributions

**Laura van der Krogt:** conceptualization, investigation, formal analysis, writing – original draft. **Lisa Story:** writing – review and editing, supervision. **Natalie Suff:** writing – review and editing, supervision. **Andrew Shennan:** conceptualization, writing – review and editing, supervision.

## Funding

This work did not receive any specific grant from funding agencies in the public, commercial, or not‐for‐profit sectors. Laura van der Krogt and the ABOVE trial are funded by Action Medical Research/Borne grant GN2967. Dr. Lisa Story is supported by an NIHR Advanced Fellowship number 301664. Dr. Natalie Suff is supported by a Wellcome Clinical Research Career Development Fellowship 222970/Z/21/Z.

## Ethics Statement

The PCN Database has been reviewed and approved by the East of Scotland Research Ethics Service REC 2 (research ethics committee reference number 16/ES/0093). All study participants provided informed consent.

## Conflicts of Interest

The authors declare no conflicts of interest.

## Supporting information


**Table S1:** Outcomes of previous pregnancy with transabdominal cerclage.

**Figure S1:** Box plot of second trimester CL in women with a ‘successful’ cerclage (delivery > 32 weeks) and cerclage failure (delivery < 32 weeks).

## Data Availability

The data that support the findings of this study are available on request from the corresponding author. The data are not publicly available due to privacy or ethical restrictions.
