# Supplementary material for: Clinical Characteristics Associated With Very Preterm Delivery Despite Transabdominal Cerclage: A Cohort Study
Source: BJOG. 2026 Feb 16;133(8):1553–60. doi: 10.1111/1471-0528.70177 (PMC13253993; doi:10.1111/1471-0528.70177)
Supplement: Supplementary file 1 — Table S1: Outcomes of previous pregnancy with transabdominal cerclage. [file BJO-133-1553-s002.docx]

| **Supplementary Table 1: Outcomes of previous pregnancy with transabdominal cerclage** | |
| --- | --- |
|  | Women with pregnancies with TAC prior to index case (n = 30) |
| Pregnancy outcome   - Early miscarriage - Mid-trimester loss - Iatrogenic PTB (<37 weeks)* - Spontaneous PTB (<37 weeks) - One term delivery - Two term deliveries - Three term deliveries | 5 (16.6%)  1 (3.3%)  1 (3.3%)  2 (6.7%)  20 (66.7%)  3 (10%)  1 (3.3%) |
| *Table 6: Pregnancy outcomes of previous deliveries, prior to index case, with TAC in situ for all women in this cohort TAC = Transabdominal cerclage, PTB = preterm birth. *Iatrogenic preterm delivery for pre-eclampsia.* | |

S2 – Supplementary Figure 1: Box plot of second trimester CL in women with a ‘successful’ cerclage (delivery >32 weeks) and cerclage failure (delivery <32 weeks)
