# Supplementary figures and images for: Clinical Characteristics Associated With Very Preterm Delivery Despite Transabdominal Cerclage: A Cohort Study
Source: BJOG. 2026 Feb 16;133(8):1553–60. doi: 10.1111/1471-0528.70177 (PMC13253993; doi:10.1111/1471-0528.70177)

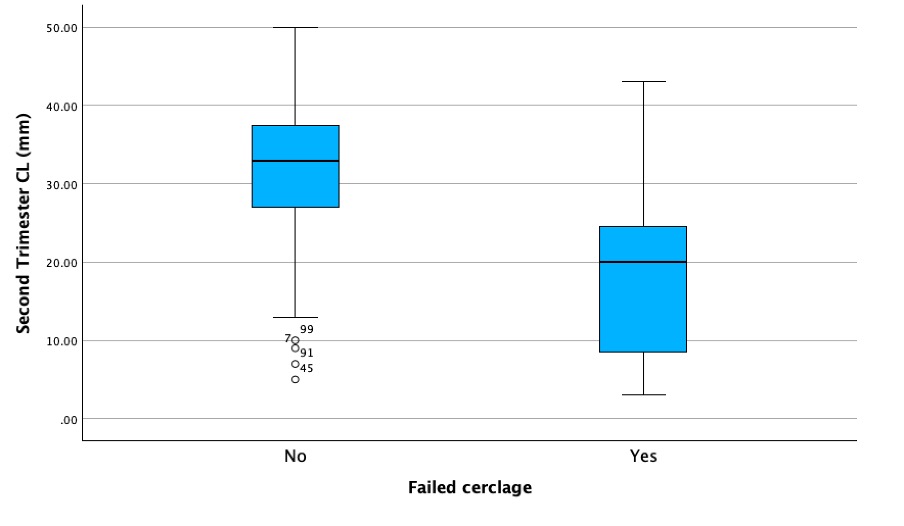

Supplement: Supplementary file 2 — Figure S1: Box plot of second trimester CL in women with a ‘successful’ cerclage (delivery > 32 weeks) and cerclage failure (delivery < 32 weeks). [file BJO-133-1553-s001.jpg]
